# Cerebral arteriopathy and its role in persistent post-COVID headache and brain fog: a quantitative study

**DOI:** 10.1007/s00406-026-02276-0

**Published:** 2026-06-13

**Authors:** Jr-Wei Wu, Shu-Ting Chen, Feng-Chi Chang, Yung-Lin Chen, Juosi Leung, Mei-Chun Chen, Jiing-Feng Lirng, Yuan-Hwa Chou

**Affiliations:** 1 Department of Neurology, Neurological Institute, Taipei Veterans General Hospital, Taipei, Taiwan; 2 College of Medicine, National Yang Ming Chiao Tung University, Taipei, Taiwan; 3 Department of Radiology, Taipei Veterans General Hospital, Taipei, Taiwan; 4 Institute of Biophotonics, National Yang Ming Chiao Tung University, Taipei, Taiwan; 5 Department of Post-Baccalaureate Medicine, College of Medicine, National Chung Hsing University, Taichung, Taiwan; 6 Department of Psychiatry, Taichung Veterans General Hospital, Taichung, Taiwan

**Keywords:** COVID-19, Brain fog, Headache, Cerebral arteriopathy

## Abstract

**Background:**

Headache and brain fog are common after COVID-19 infection, and inflammation and vasculopathy might be the potential underlying mechanisms. This study aimed to delineate the extent and impact of cerebrovascular involvement in patients with persistent long-COVID syndrome in a large Asian cohort.

**Methods:**

We prospectively collected data from patients with persistent (≥ 3 months) post-COVID headache or brain fog, and controls were free from neurological symptoms within 3 months after COVID-19 resolution. The anterior and posterior Focal Cerebral Arteriopathy Severity Score (FCASS) was used to assess the severity of arteriopathy and was modified to account for bilateral involvement (total score: anterior: 0–40; posterior: 0–52). Symptom severity was assessed using monthly headache days, Migraine Disability Assessment (MIDAS), and brain fog severity scale (0–10).

**Results:**

A total of 108 patients (M: F = 27:81) were divided into four groups based on symptoms categories. The presence of more symptom categories was associated with higher anterior-FCASS (combined 6.6 [2.1] vs. headache 3.6 [1.6] vs. brain-fog 4.7 [1.7] vs. control 0.8 [0.6], *p* < 0.001 by one-way ANOVA) and a posterior-FCASS (combined 5.5 [2.2] vs. headache 4.2 [1.6] vs. brain-fog 4.4 [2.3] vs. control 0.8 [0.8], *p* < 0.001 by one-way ANOVA). Pure brain- fog was more likely have predominance of anterior circulation involvement than pure headache (80.8% vs. 48.0%, *p* = 0.020), but the severity of brain fog was correlated with only the posterior-FCASS (Pearson’s *r* = 0.338, *p* = 0.009) rather than the anterior-FCASS (Pearson’s *r* = 0.173, *p* = 0.195).

**Conclusions:**

Anterior circulation arteriopathy and hypoperfusion may underlie persistent post-COVID brain fog. However, post-COVID headaches may not depend solely on changes in intracranial vessels.

## Introduction

Coronavirus disease 2019 (COVID-19) is an infectious disease caused by severe acute respiratory syndrome coronavirus type 2 (SARS-CoV-2), and caused a devastating global pandemic. Moreover, COVID-19 patients may experience neuropsychiatric symptoms after infection, such as headache, dizziness, mood changes, fatigue, and cognitive problem [1]. These symptoms are collectively called long-COVID or post-acute sequelae of COVID-19 (PASC) [1, 2]. Long-COVID or PASC is common, affecting approximately 31–69% of COVID-19 patients in different studies [3]. Among these neuropsychiatric symptoms, headache is one of the common symptoms after the COVID-19 infection [4]. One study of the original strain revealed that ~ 65% of patients reported headaches during the acute phase of COVID-19 and 15% report continuous headache [5], and migraine-like headache was the most common phenotype (51%) [5]. On the other hand, several patients reported experiencing ‘brain fog’ after the COVID-19 infection, namely cognitive impairment, fatigue, and depression, which causes a substantial burden on patients’ quality of life. Moreover, some patients might develop headaches with other neuropsychiatric symptoms (i.e., brain fog) after COVID-19 infection [4], and the pathophysiological and clinical implications of this subgroup warrant further investigations.

The pathophysiology of these neurological long-COVID symptoms includes inflammation and vasculopathy due to endothelial dysfunction and coagulopathy [6–8]. Other studies have also reported low cortisol production and hypothalamus–pituitary–adrenal axis dysfunction after one year of long COVID symptoms [9]. Also, a study revealed reduced gray matter thickness over the orbitofrontal cortex and parahippocampal gyrus [10], as well as a reduction in overall brain volume, among patients with cognitive impairment after COVID-19 [10]. There have also been reports of cerebral vascular damage and cerebral arteriopathy during or after COVID-19 infection [11–14]. Hence, the prevalence and occurrence of arteriopathy after COVID-19, as well as the relationship of arteriopathy with neurological long-COVID symptoms, warrant further investigations. In this study, we aimed to analyze the associations between different post-COVID neuropsychiatric symptoms, namely, brain fog and headache, and the severity of cerebral arteriopathy.

## Methods

### Study population

We prospectively recruited patients with persistent post-COVID headaches and/or brain fog from the neurological clinic of the Taipei Veterans General Hospital between August 2022 and August 2024. All post-COVID infected patients completed a structuralized questionnaire for post-COVID neuropsychiatric symptoms. Post-COVID headache was diagnosed by headache specialists according to the International Classification of Headache Disorders, 3rd edition (ICHD-3) [15]. In ICHD-3, no headache diagnoses included headaches specifically after the ‘COVID-19’ infection [15]. Therefore, this study recruited patients with post-COVID headache who fulfilled ‘9.2.2 Headache attributed to systemic viral infection’ criterion of the ICHD-3, and the headache developed or worsened within four weeks after COVID-19 infection [15]. On the other hand, whilst ‘brain fog’ is often used in descriptions of cognitive symptoms after COVID-19 infection, there are no consensus diagnostic criteria for brain fog [16]. In this study, ‘brain fog’ was operationally defined using symptom descriptors originally proposed by Ross et al. (2013) to characterize cognitive complaints in patients with postural orthostatic tachycardia syndrome (POTS) [17]. To ensure relevance to post-COVID-19 populations, these descriptors were restricted to those overlapping with the four most frequently reported symptoms identified in large-scale analyses of post-COVID brain-fog [18].

In this study, we recruited patients who developed brain fog within four weeks after COVID-19 infection. Moreover, these patients had at least 2 out of 4 most common brain fog symptoms, including 1) forgetfulness, 2) excessive cognitive effort (or difficulty thinking), 3) difficulty concentrating, and 4) difficulty communicating or word-finding problems [16, 19]. In this study, ‘persistent’ post-COVID headache and/or brain fog was defined as symptoms occurring for ≥ 15 days per month for at least 3 months.

### Inclusion and exclusion criteria

In this study, patients were designated into four groups: post-COVID headache, post-COVID brain-fog, post-COVID combined symptoms, or post-COVID control group. Patients in the ‘combined group’ were required to meet the criteria for post-COVID headache and post-COVID brain-fog.

#### Post-COVID headache group

The inclusion criteria for patients with persistent post-COVID headache were as follows: (1) diagnosis by headache specialists based on the ICHD-3 diagnostic criteria (‘9.2.2 headache attributed to systemic viral infection’ within four weeks after a documented COVID-19) [15]; (2) headache frequency ≥ 15 days per month for ≥ 3 months; (3) an age between 20 and 65 years; and (4) the ability to undergo magnetic resonance imaging (MRI) without contraindications.

The exclusion criteria were as follows (1) the presence of other secondary headache disorders according to the ICHD-3 [15]; (2) a history of cerebrovascular disorders before COVID-19 infection; and (3) a history of systemic disorders, including uncontrolled hypertension, diabetes, chronic kidney disease, autoimmune disease, cirrhosis, and malignancy; (4) a history of head or neck malignancies or infection that may alter the paranasal sinus structure; and (5) pregnancy or lactation.

#### Post-COVID brain-fog group

The inclusion criteria for patients with persistent post-COVID brain-fog were as follows: (1) the development of brain fog symptoms within four weeks after a documented COVID-19 infection and the presence of at least 2 of the following 4 symptoms: forgetfulness, excessive cognitive effort (or difficulty thinking), difficulty concentrating, and difficulty communicating or word-finding problems [11]; (2) a frequency of brain fog ≥ 15 days per month for ≥ 3 months; (3) an age between 20 and 65 years; and 4). the ability to undergo MRI without contraindications.

The exclusion criteria included the following: (1) identified secondary causes of brain fog other than COVID-19; (2) a history of cerebrovascular disorders before COVID-19 infection, (3) a history of systemic disorders, including uncontrolled hypertension, diabetes, chronic kidney disease, autoimmune disease, cirrhosis, and malignancy; (4) a history of head or neck malignancies or infection that may alter the paranasal sinus structure; and (5) pregnancy or lactation.

#### Post-COVID control group

The inclusion criteria for patients with persistent post-COVID headache were as follows: (1) a history of documented COVID-19 infection, (2) no headache for ≥ 3 months after the COVID-19 infection, (3) no brain fog or other cognitive symptoms for ≥ 3 months after the COVID-19 infection; (4) an age between 20 and 65 years; and (5) the ability to undergo MRI without contraindications.

The exclusion criteria were as follows (1) the presence of other secondary headache disorders according to the ICHD-3 [15]; (2) identified secondary causes of brain fog other than COVID-19; (3) a history of cerebrovascular disorders before COVID-19; (4) a history of systemic disorders, including uncontrolled hypertension, diabetes, chronic kidney disease, autoimmune disease, cirrhosis, and malignancy; (5) a history of head or neck malignancies or infection that may alter the paranasal sinus structure; and (6) pregnancy or lactation.

### Brain neuroimaging acquisition

All participants in this study underwent MRI on a 1.5T scanner (Optima™ MR450w GEM, GE Healthcare, United States) using a standard eight-channel head coil. The following sequences were performed with their respective technical parameters. Axial T2-weighted images were acquired with a repetition time (TR)/echo time (TE) of 6810/110 ms, number of excitations (NEX) of 1, a matrix size of 512 × 384, a field of view (FOV) of 256 × 256 mm, and a slice thickness of 5 mm. Axial T1-weighted images were obtained with TR/TE = 9.7/3.8 ms, NEX = 2, a matrix size of 512 × 384, an FOV of 256 × 256 mm, and a slice thickness of 1.0 mm. Three-dimensional time-of-flight (3D-TOF) MR angiography (MRA) of the brain was performed with a TR/TE = 26/6.8 ms, a matrix size of 320 × 224, an FOV of 200 × 200 mm, a flip angle of 20 degrees, and 5 slabs. MR angiograms were obtained from different viewing directions were obtained using a maximum-intensity projection (MIP) algorithm.

### Brain fog severity measurements

In this study, we employed a self-reported brain fog severity score described by Novak et al. (2022), an 11-point scale ranging from 0 (no brain fog) to 10 (severe, disabling brain fog, constantly present), to assess the severity of brain fog following COVID-19 infection [19].

### Evaluation and grading of intracranial vessels

**Fig. 1:**
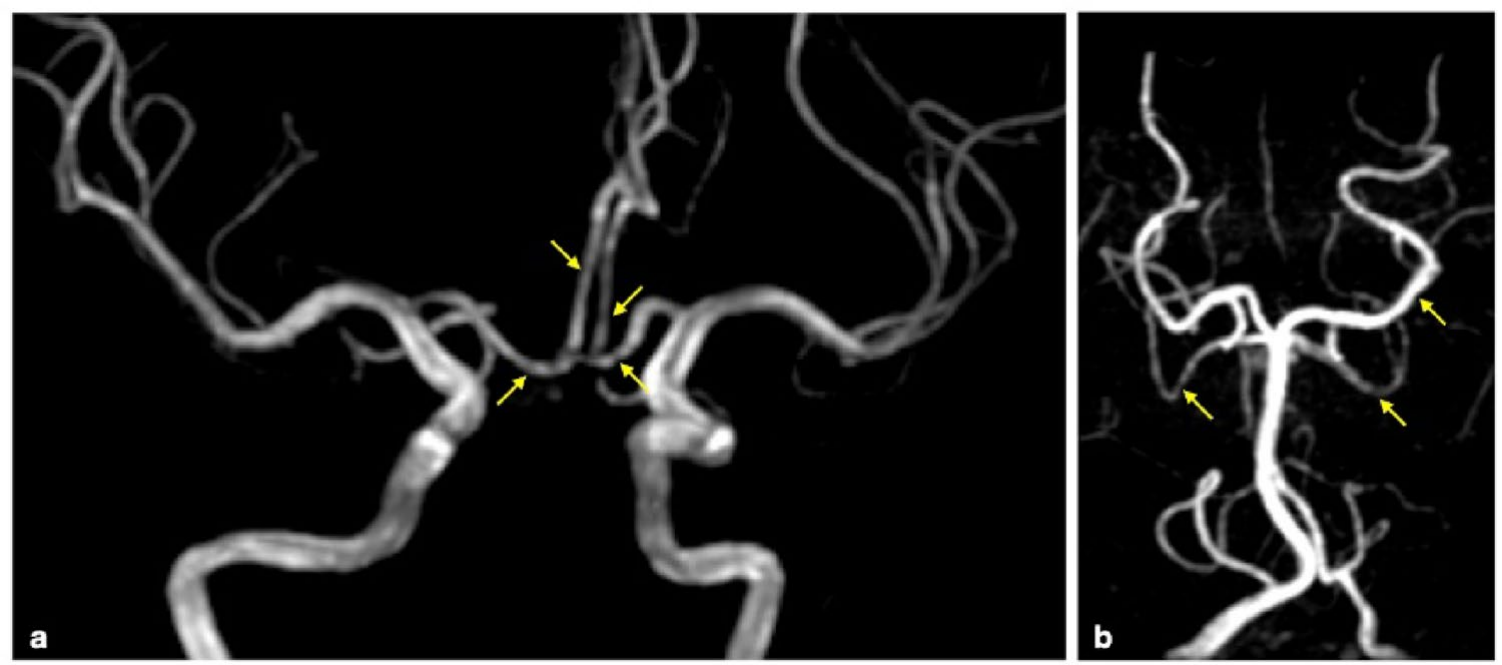
Measurement of anterior- and posterior-FCASS. TOF-MRA MIP images (**a**, **b**) show multiple vessel abnormalities (arrows): **a** Banding of A2 segment of the bilateral anterior cerebral arteries; stenosis at A1 segment of the anterior cerebral artery on the right and left (anterior-FCASS = 6); **b** Banding of P2 segment of the left posterior cerebral artery; stenosis of the bilateral superior cerebellar arteries (posterior-FCASS = 7)

The modified anterior and posterior Focal Cerebral Arteriopathy Severity Score (FCASS) was used to assess the severity of arteriopathy [9, 20]. The anterior-FCASS assesses five arterial segments, including supraclinoid internal carotid artery, A1 and A2 of the anterior cerebral artery (ACA); and M1, and M2 of the middle cerebral artery (MCA) [9]. The posterior-FCASS accesses intracranial vertebral artery, basilar artery (BA), P1 and P2 segments of the posterior cerebral artery, the superior cerebellar artery (SCA), the anterior inferior cerebellar artery (AICA), and the posterior inferior cerebellar artery (PICA) [20]. All segments are assigned a severity score: 0, no involvement; 1, irregularity or banding with no stenosis; 2, stenosis with a < 50% reduction in diameter; 3, stenosis with a > 50% reduction in diameter; and 4, occlusion. For small arteries (A2, M2, SCA, PICA, and AICA), any stenosis of the segments is scored as 3 (with no option for a score of 2) [9, 20]. Consequently, these segments are classified into one of only four categories (scores of 0, 1, 3, or 4). In this study, we modified the anterior and posterior-FCASS, which were originally developed to analyze “unilateral and unifocal” focal cerebral arteriopathy [9, 20]. To account for bilateral involvement in our participants, we modified the original score (anterior: 0–20; posterior: 0–28) to account for bilateral involvement (anterior: 0–40; posterior: 0–52), as shown in Fig. 1.

### Evaluating the predominance of anterior or posterior circulation involvement

We calculated the percentage by dividing each participant’s FCASS by the maximum score (anterior-FCASS: 40; posterior-FCASS: 52) to determine the predominant circulation involvement (anterior or posterior). We compared this percentage between anterior and posterior circulation, and a higher percentage was considered predominant.

### Standard protocol approvals, registrations, and patient consents

The study protocol was approved by the Institutional Review Board of the Taipei Veterans General Hospital (2022-07-024BC).

### Statistics

SPSS version 22.0 (SPSS Inc., Chicago, Illinois, USA) was used for the data analyses. The sex distribution of the four groups was analyzed by chi-square tests, and the mean ages of the four groups was analyzed by one-way ANOVA. The comparisons of the clinical profiles and headache-associated symptoms between the two groups with post-COVID headaches (headache only and headache + brain-fog) were performed using t-tests or chi-square tests as appropriate. The comparison of clinical profiles and symptomatology of the two groups with post-COVID brain-fog was analyzed by using t-tests or chi-square tests as appropriate. *P* < 0.05 was applied to determine significance for demographics and clinical profiles.

Comparisons of FCASS (anterior or posterior) among the four groups were performed by one-way ANOVA with Tukey’s post hoc test, and paired comparisons between groups were performed by t-tests, with a significance threshold of *p* < 0.05. The correlation between symptom severity (headache frequency and brain-fog severity score) and FCASS (anterior or posterior) was analyzed by Pearson’s r. The Bonferroni correction for multiple comparisons was applied, and *p* < 0.0125 was considered to indicate significance (corrected for four pairwise comparisons: *p* = 0.05/4 tests = 0.0125). In the exploratory analyses, we also compared the predominance of anterior or posterior circulation involvement in patients with persistent neurological post-COVID symptoms, which included brain-fog (+) vs. brain-fog (-), headache (+) vs. headache (-), and pure headache vs. pure brain-fog. These comparisons were performed by chi-square analysis with a significance threshold of *p* < 0.05.

## Results

### Patients

Between August 2022 and August 2024, a total of 108 patients (M: F = 27: 81) had history of COVID-19 infection were recruited and complete the study. The mean (SD) age of the participants who completed the study was 37.5 [12.0] years. Patients were designated into four groups, including headache (*n* = 25), brain-fog (*n* = 26), combined (headache and brain-fog, *n* = 32), and controls groups (*n* = 25). Among patients with post-COVID headache, 89.5% (51/57) had a migraine-like headache, whereas 10.5% (6/57) had a tension-type headache (TTH)-like presentation. The distribution of headache phenotypes did not differ significantly between the two groups (Fisher’s exact test, *p* = 0.686). There were no differences in age or sex among the groups, and the demographics and clinical profiles are shown in Table 1.

**Table 1:** Demographics and clinical profiles of post-COVID patient groups and controls

Demographics	Headache (*n* = 25)	Brain-fog (*n* = 26)	Combined symptoms (*n* = 32)	Controls (*n* = 25)	*p* value *
Females, sex, n (%)	20 (80.0%)	20 (76.9%)	24 (75%)	17 (68.0%)	0.792
Age, years, mean (SD)	37.7 (13.2)	39.0 (10.0)	37.1 (13.2)	36.2 (11.7)	0.868
Headache frequency, days/months, mean (SD)	17.6 (4.6)	NA	19.5 (5.9)	NA	0.183
Brain-fog severity, mean (SD)	NA	2.7 (2.7)	4.3 (2.1)	NA	0.016*
MIDAS, mean (SD)	21.6 (24.9)	NA	31.8 (39.4)	NA	0.265
Headache phenotypes					
Migraine-like, n (%)	23(92.9%)	NA	28 (87.5%)	NA	0.686
TTH-like, n (%)	2 (8.0%)		4 (12.5%)		
Nausea, n (%)	16 (64.0%)	NA	21 (65.6%)	NA	0.898
Vomiting, n (%)	10 (40%)	NA	21 (65.6%)	NA	0.054
Photophobia, n (%)	8 (32.0%)	NA	15 (46.9%)	NA	0.256
Phonophobia, n (%)	11 (44.0%)	NA	22 (68.8%)	NA	0.060
Forgetfulness, n (%)	NA	22 (84.6%)	31 (96.9%)	NA	0.098
Difficulty thinking, n (%)	NA	14 (53.8%)	19 (59.4%)	NA	0.672
Difficulty concentrating, n (%)	NA	21 (80.8%)	26 (81.3%)	NA	0.963
Difficulty communicating or word-finding problems, n (%)	NA	16 (61.5%)	20 (62.5%)	NA	0.940
Mental fatigue, n (%)	NA	10 (38.5%)	13 (40.6%)	NA	0.867
Mental slow, n (%)	NA	9 (34.6%)	19 (59.4%)	NA	0.061
Easily distracted, n (%)	NA	9 (34.6%)	15 (46.9%)	NA	0.346
Annoying, n (%)	NA	8 (30.8%)	13 (40.6%)	NA	0.437
Sleepiness, n (%)	NA	11 (42.3%)	18 (56.3%)	NA	0.291

*Differences between continuous variables were analyzed by paired t-test or one-way ANOVA, and the differences between categorical variables were analyzed by the chi-square test or Fisher’s exact test, as appropriate; *p* < 0.05 was considered statistically significant

### Severity of cerebral arteriopathy and symptomatology

**Fig. 2:**
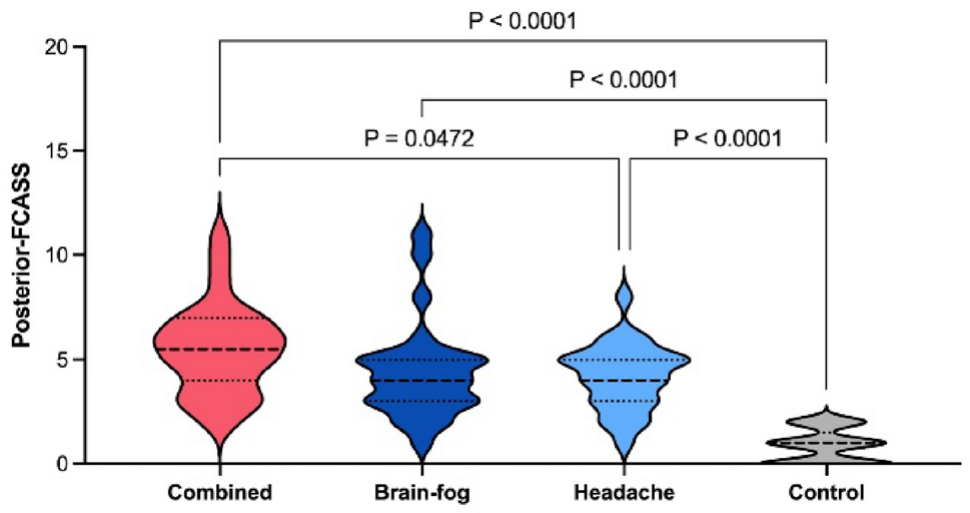
Anterior-FCASS in each group. The paired comparisons between groups were performed by t-tests, with a significance threshold of *p* < 0.05

**Fig. 3:**
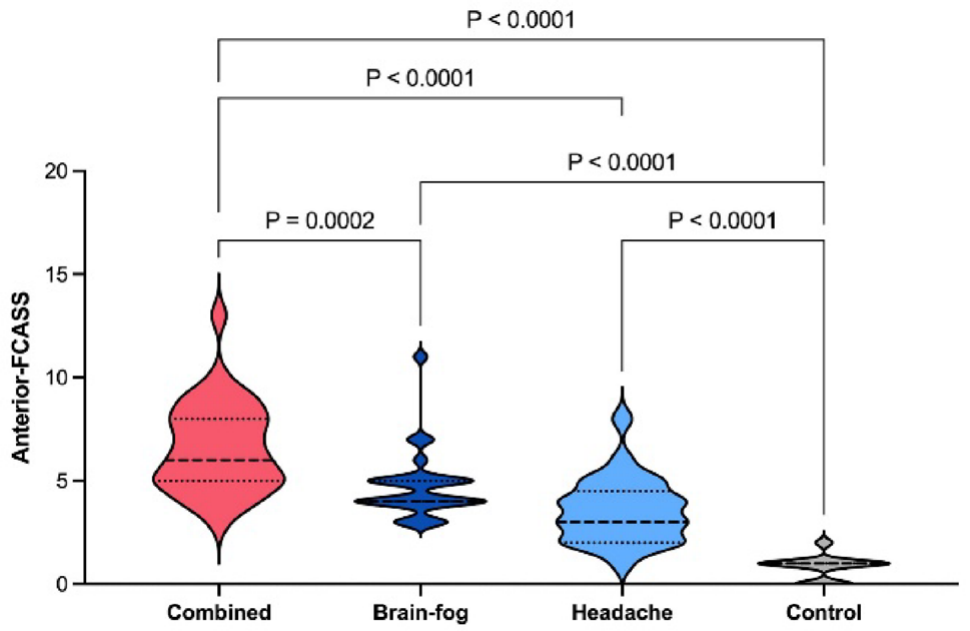
Posterior-FCASS in each group. The paired comparisons between groups were performed by t-tests, with a significance threshold of *p* < 0.05

There was no association between age and severity of cerebral arteriopathy (anterior- FCASS: Pearson *r* = 0.020, *p* = 0.833; posterior-FCASS: Pearson *r* = 0.110, *p* = 0.256). Additionally, sex was not associated with severity of arteriopathy (anterior FCASS: *p* = 0.166; posterior FCASS: *p* = 0.844). The presence of more symptom categories was associated with a higher anterior-FCASS (combined 6.6 [2.1] vs. headache 3.6 [1.6] vs. brain-fog 4.7 [1.7] vs. control 0.8 [0.6], *p* < 0.001 by one-way ANOVA with Tukey’s post hoc test) (Fig. 2). In terms of posterior circulation, the posterior-FCASS (bilateral score, range 0–52) was also higher in patients with more symptom categories (combined 5.5 [2.2] vs. headache 4.2 [1.6] vs. brain-fog 4.4 [2.3] vs. control 0.8 [0.8], *p* < 0.001 by one-way ANOVA with Tukey’s post hoc test), and patients with any or combined symptoms were significantly higher than controls (Fig. 3).

We further separately analyzed the associations between symptoms (brain-fog and headache) and FCASS (anterior and posterior). In patients with brain-fog (combined group + brain-fog group, *n* = 58), the brain-fog severity score was correlated with posterior-FCASS (Pearson’s *r* = 0.338, *p* = 0.009), but not anterior-FCASS (Pearson’s *r* = 0.173, *p* = 0.195). Among patients with headache (combined group + headache group, *n* = 57), there was no correlation between headache frequency and anterior-FCASS (Pearson’s *r*=−0.059, *p* = 0.660), nor posterior-FCASS (Pearson’s *r* = 0.196, *p* = 0.144).

### Exploratory: distribution of arteriopathy on MR angiography

Among patients with persistent neurological post-COVID symptoms (*n* = 83), 73.5% had predominant anterior-circulation involvement. Patients with brain-fog tended to predominantly involve anterior circulation (% of predominant anterior circulation involvement, brain-fog + vs. brain-fog-: 84.5% vs. 48.0%, *p* = 0.001). On the other hand, the presence or absence of headache was not associated with differences in the prevalence of predominant anterior circulation involvement (% of predominant anterior circulation involvement, headache + vs. headache-: 70.2% vs. 80.8%, *p* = 0.310). After excluding patients with combined symptoms, we found patients with pure brain fog (*n* = 26) had a higher percentage of predominant anterior circulation involvement than those with pure headache (*n* = 25) (80.8%, 48.0%, *p* = 0.020).

## Discussion

In this study, we found that cerebral arteriopathy was associated with patients with persistent neurological symptoms after COVID-19 infection. Patients with post-COVID brain-fog tended to have more prominent anterior circulation involvement, whereas those with post-COVID headaches showed similar involvement between the anterior and posterior circulation. In patients with brain fog, self-reported brain-fog severity was positively correlated with the severity of cerebral arteriopathy in the posterior circulation. However, we did not observe such a correlation between headache frequency and the severity of cerebral arteriopathy in either the anterior or posterior circulation.

A key strength of this study is the use of a quantitative scoring system to assess severity. Additionally, we examined the distribution of arteriopathy and its association with clinical symptoms and severity. In this study, we found that brain-fog tended to have anterior circulation involvement, while the severity of posterior circulation arteriopathy was correlated with the severity of brain fog. These findings indicate that the brain fog is the dysfunction across multiple cognitive domains, and the hypoperfusion of cerebral cortex is crucial for the development of brain fog [21–23]. The involvement of the posterior circulation appears to have an additive effect on brain-fog severity. A previous perfusion study analyzing patients with persistent cognitive complaints after COVID-19 infection revealed that cerebral hypoperfusion was more prominent in the bilateral frontal, temporal, and parietal regions [23]. Moreover, a SPECT study demonstrated reduced cerebral blood flow in the lateral prefrontal and sensorimotor cortices in patients with cognitive dysfunction and postural orthostatic tachycardia syndrome following COVID-19 infection. These findings are concordant with our observation of more pronounced hypoperfusion in the anterior circulation among patients with post-COVD cognitive deficits [24].

The presence of cerebral arteriopathy in our patients with post-COVID headache persisting for ≥ 3 months may reflect prolonged post-infectious inflammation, which has been proposed as a common trigger for headache exacerbation [25]. In addition, previous studies have shown that flu-like illness is a risk factor for the abrupt transition from episodic to chronic headache disorders, or the development of new daily persistent headache (NDPH)-like headaches [26, 27]. However, in the pure post-COVID headache group, there was similar involvement of the anterior and posterior circulations (48% vs. 52%). This observation suggests that post-COVID headache may be primarily driven by inflammatory mechanisms and activation of meningeal nociceptors [28, 29], rather than by the distribution of cerebral arteriopathy in the anterior or posterior circulation; consequently, intracranial vascular changes alone are insufficient to fully explain the development of post-COVID headache. Previous studies have shown that elevated CGRP levels and activation of the trigeminovascular pathway are more strongly associated with post-COVID headaches, and some patients may benefit from CGRP-targeted therapy [30, 31]. Our findings suggest that even in patients with post-COVID headaches resembling migraine, their underlying cause may not be attributed to changes in intracranial vessels or hypoperfusion.

Our study provides insights into the pathophysiology of long-COVID neurological symptoms. According to our findings, the cerebral arteriopathy could be found in both post-COVID headache and brain-fog groups, highlighting the importance of cerebral vascular condition work-ups in this population. The concept of cerebral focal arteriopathy was initially proposed as one of the most common causes of pediatric stroke following viral infection, but focal cerebral arteriopathy has also been reported among young adults [32, 33]. There were also differences in cerebral arteriopathy between pediatric stroke patients and patients with persistent long COVID in the present study. In pediatric patients, focal cerebral arteriopathy is mostly unilateral, and varicella zoster virus (VZV) is a long-established cause of focal cerebral arteriopathy [9, 34, 35], whereas in our long-COVID patients, it typically involves bilateral regions [36]. In both conditions, the arteriopathy is presumed to be inflammatory in origin that following a viral infection. Several studies have reported an increased risk of vascular injury during and after COVID-19 infection [37, 38]. Additionally, research has proposed possible mechanisms underlying long-COVID neurological symptoms, including endothelial dysfunction, hypercoagulable states, and clot formation [39, 40]. These mechanisms may underlie our neuroimaging findings of cerebral arteriopathy.

Our research findings have important clinical implications. Although headache and brain fog are not uncommon following COVID-19 infection, brain neuroimaging should be considered in patients with persistent symptoms lasting more than three months (symptom frequency ≥ 15 days per month for ≥ 3 months) to identify post-COVID cerebrovascular complications, such as cerebral arteriopathy or vasospasm. Early identification of these abnormalities may assist clinicians in initiating appropriate management strategies. Notably, although no patients in our cohort presented with thunderclap headache, beading pattern of the cerebral arteries accompanied by focal stenosis on magnetic resonance angiography was observed in 24 out of 83 patients (28.9%) with post-COVID headache and/or brain-fog, suggesting the concomitant vasospasm. This observation is in line with previous reports demonstrating that cerebral vasospasm and arteriopathy may be present in the same patient [41]. Taken together, these findings indicate that evaluation for cerebrovascular involvement should not be restricted to patients with post-COVID headache who present with traditional ‘red flags’ for secondary headache (e.g., thunderclap headache). Patients with persistent migraine-like or TTH-like headache may also warrant neuroimaging assessment. In patients with cerebral arteriopathy accompanied by vasospasm, treatment with calcium channel blockers, such as nimodipine, may be a reasonable therapeutic option [42, 43].

Our study has several limitations. First, our study population included patients with persistent (≥ 15 symptom days/month for ≥ 3 months) neurological symptoms after the resolution of COVID-19 infection. Thus, our findings represent only a subgroup of patients with severe postinfectious symptoms. Patients with a relatively shorter duration of post-COVID symptoms may have a different underlying pathophysiology. Nevertheless, our study identified potential new neuroimaging findings in this intractable subgroup, providing insight for future research. Second, as this was a matched case-control study, the number of patients in each group does not reflect the actual distribution of symptom groups in the broader population. This design was chosen to quantitatively compare the anterior- and posterior-FCASS, making it essential to have an equal distribution across symptom groups for neuroimaging score comparisons. Third, we did not compare brain MRAs before and after COVID-19 infection, as it is not feasible to recruit patients who will develop persistent post-COVID symptoms and had undergone brain MRI prior to the pandemic. However, we excluded patients aged ≥ 65 years and those with vascular risk factors to minimize potential confounding due to pre-existing cerebrovascular pathologies. Finally, we did not use protocols such as vessel wall imaging to differentiate the exact mechanisms of the observed vascular lesions in our study. Since this study was exploratory, the main purpose was to describe the findings in intracranial arteries among patients with persistent long-COVID. Future studies are needed to further explore the nature of these changes in cerebral arteries.

## Conclusions

Cerebral arteriopathies are observed in patients with persistent post-COVID brain fog or headache. Hypoperfusion in the anterior circulation may underlie post-COVID brain fog, with posterior circulation involvement having an additive effect. The development of persistent post-COVID headaches, however, may not solely depend on changes in intracranial vessels.

## Data Availability

Data would be available on reasonable request.
